# Fecal ACE and ACE2 Activities Reflect Intestinal Shedding and Microbiota Modulation of Renin–Angiotensin System

**DOI:** 10.3390/biology15100776

**Published:** 2026-05-13

**Authors:** Mariana Ferreira-Duarte, Clara Quintas, Joana Bom, Ana Lúcia Ribeiro, Marília Pereira, Michael Bader, Natalia Alenina, Kenneth E. Bernstein, Ellen A. Bernstein, Teresa Sousa, Fernando Magro, Margarida Duarte-Araújo, Lilian Caroline Gonçalves Oliveira, Dulce Elena Casarini, Manuela Morato

**Affiliations:** 1 Laboratory of Pharmacology, Department of Drug Sciences, Faculty of Pharmacy of the University of Porto (FFUP), 4050-313 Porto, Portugal; 2 LAQV@REQUIMTE, University of Porto, 4050-313 Porto, Portugal; 3 UCIBIO@REQUIMTE, University of Porto, 4050-313 Porto, Portugal; 4 Gulbenkian Institute for Molecular Medicine, Oeiras Site, 2780-156 Oeiras, Portugal; 5 Max Delbrück Center (MDC), 13092 Berlin, Germany; 6 German Center for Cardiovascular Research (DZHK), Partner Site Berlin, 10785 Berlin, Germany; 7 Charité Universitätsmedizin Berlin, 10117 Berlin, Germany; 8 Institute for Biology, University of Lübeck, 23562 Lübeck, Germany; 9 Department of Biomedical Sciences, Cedars-Sinai Medical Center, Los Angeles, CA 90048, USA; 10 Department of Pathology, Cedars-Sinai Medical Center, Los Angeles, CA 90048, USA; 11 RISE-Health, Department of Biomedicine, Unit of Pharmacology and Therapeutics, Faculty of Medicine, University of Porto (FMUP), 4200-450 Porto, Portugal; 12 CINTESIS@RISE, Faculty of Medicine of the University of Porto (FMUP), 4200-450 Porto, Portugal; 13 Department of Immuno-Physiology and Pharmacology, School of Medicine and Biomedical Sciences (ICBAS), University of Porto, 4050-313 Porto, Portugal; 14 Department of Medicine, Discipline of Nephrology, Escola Paulista de Medicina, Universidade Federal de São Paulo (EPM/UNIFESP), São Paulo 04023-900, Brazil

**Keywords:** angiotensin-converting enzyme (ACE), angiotensin-converting enzyme 2 (ACE2), ACE-like enzyme, feces, microbiota, shedding

## Abstract

Angiotensin-converting enzymes (ACE and ACE2) are key components of the renin–angiotensin–aldosterone system (RAAS) and are present in the gastrointestinal tract and intestinal content, preserving their catalytic activity, and may interact with the gut microbiota. The present study aimed to determine the origin of fecal ACE and ACE2 activity. Fecal pellets from germ-free, ACE and ACE2 knockout (KO) mice, and from the corresponding controls were analyzed using fluorimetric enzyme activity assays. ACE activity was assessed using Hippuryl-His-Leu and Z-Phe-His-Leu as substrates; ACE2 activity was assessed using Mca-APK (Dnp), with and without the ACE2 inhibitor MLN-4760. Germ-free mice showed increased fecal ACE and ACE2 activity compared to controls. ACE2-KO mice lacked fecal ACE2 activity, whereas ACE activity was unaffected. In ACE-KO mice, fecal ACE activity was reduced, but not abolished, while ACE2 activity remained similar to controls. In ACE C- and N-domain KO mice, ACE activity was similar to controls, and inhibition with captopril completely abolished fecal ACE activity using Hippuryl-His-Leu, but not Z-Phe-His-Leu, in those animals. These findings indicate that fecal ACE and ACE2 activity results from combined intestinal shedding and microbiota-related mechanisms, supporting a modulatory role of the gut environment on luminal RAAS activity.

## Simple Summary

Angiotensin-converting enzyme and angiotensin-converting enzyme 2 are important regulators of the renin–angiotensin–aldosterone system, a hormonal pathway classically associated with blood pressure control. However, these enzymes are also present in the gastrointestinal tract, and we have previously shown that they are also present in the feces, raising the question of where these enzymes originate from. We aimed to investigate whether fecal enzymes come from the intestinal wall, from the gut microbiota, or from both sources. Using fecal samples from germ-free mice and genetically modified mice lacking angiotensin-converting enzyme or angiotensin-converting enzyme 2, we found that fecal activity from both enzymes reflects both intestinal release and additional enzyme activity likely influenced by gut microorganisms. These findings reveal that the intestinal environment can shape local renin–angiotensin–aldosterone system activity and suggest that fecal angiotensin-converting enzyme and angiotensin-converting enzyme 2 may become useful non-invasive markers of gut physiology and host–microbiota interactions.

## 1. Introduction

Angiotensin-converting enzyme (ACE) and ACE2 are two essential membrane-bound enzymes of the renin–angiotensin–aldosterone system (RAAS). Although they are generally associated with the cardiovascular system, all RAAS components, including ACE and ACE2, have been found to be present in the gastrointestinal tract [1]. Moreover, we have recently reported that ACE and ACE2 are catalytically active along the intestinal content of Wistar Han rats and that the catalytical activity of both ACE and ACE2 in the intestinal content was higher than that found in the corresponding intestinal region [2]. The presence of ACE has been reported in the ileal content of ostomized patients [3] and in the stools of healthy individuals and celiac patients [4]. But, to our knowledge, our group was the first to report the presence of both ACE and ACE2 activities in the rat intestinal content [2]. This data opens a new view on the state-of-the-art on RAAS and needs to be further characterized. Particularly, it is important to understand the source of both enzymes in the fecal content, with shedding from the intestinal wall and the involvement of the gut microbiota emerging as pivotal factors.

ACE and ACE2 shedding from cell membranes into their soluble isoforms has already been reported [5]. In fact, ACE isoforms of different molecular weights have been described in rats [6], in the urine of infants [7,8], hypertensive individuals [9,10] and in normotensive subjects with a family history of essential hypertension [11]. Additionally, ACE2 isoforms have also been described in the human airway epithelia [12] and bronchial epithelial cells [13], as well as in in vitro assays [5,14]. Wysocki and colleagues have shown that both urine and kidney lysates from mice can produce shorter ACE2 isoforms that are enzymatically active [15]. So, it seems plausible to assume that shedding may also occur in the intestinal wall. The ACE sheddase has not yet been identified, although it has been suggested to be a metalloproteinase [7] or a serine protease [16]. ACE2 shedding can be mediated by TMPRSS2 and ADAM-17, although only ADAM-17 sheddase activity results in extracellular soluble ACE2 [17,18]. ADAM-17 has been found in the colonic mucosa [19] and colonic epithelial cells [20].

The interplay between the RAAS and the microbiota has just started to be explored, and both ACE and ACE2 may regulate or be regulated by the microbiota [21]. It has been reported that ACE inhibitors may be produced during bacterial fermentation processes [22,23,24,25,26]. Contrarily, the microbiota also seems to catabolize ester ACE inhibitors, lowering their antihypertensive effect [27]. ACE2 is required for the expression of the neutral amino acid transporter in the intestine [28], being an important regulator of microbiota composition. Additionally, the microbiota reduces intestinal levels of ACE2, since germ-free mice were found to have higher mRNA levels of intestinal ACE2 than conventional mice [29], and reconstitution of gut microbiota reduced ACE2 expression in germ-free colonized rodents compared to germ-free rodents [30,31]. Interestingly, bacteria-derived ACE-like [32] and ACE2-like [33] enzymes have been described.

Considering the above, the aim of this study was to assess if ACE and/or ACE2 activity observed in the rat feces is a result of shedding from the intestinal wall or a product of the intestinal microbiota.

## 2. Materials and Methods

### 2.1. Ethics

Animals were housed and maintained in accredited animal facilities under institutional and national regulations governing animal welfare. Animals were not particularly ascribed to this study (3R’s—Reduction) and since only feces were used, no additional project-specific procedures beyond non-invasive fecal collection were performed. As such, no authorization from ethics committees was needed. This study followed the ARRIVE guidelines for reporting experiments [34].

### 2.2. Animals

Fresh cage fecal pellets from germ-free, ACE or ACE2 knockout (KO) mice and their corresponding control mice were used to assess the role of the gut microbiota or intestinal ACE or ACE2 shedding as a source of ACE and/or ACE2 in the feces.

#### 2.2.1. Germ-Free Animals

C57BL/6J (Charles River # 680C57BL/6J) mice were produced and maintained in germ-free conditions, at the Axenic/Gnotobiology Facility of Instituto Gulbenkian de Ciência (IGC—Lisboa, Portugal). For axenic colony management and maintenance of stock animals, rigid isolators, transfer chambers and containers were used and equipped with the Double Door Rapid Transfer Port (DPTE^®^), a user-friendly and safe connecting system for introducing and removing equipment and other materials to and from sterile isolators. Routine microbiological monitoring and 16S qPCR were used to assure the germ-free status of the animals. Age-matched specific-pathogen-free (SPF) C57BL/6J mice from the SPF Facility of IGC were used as controls. Both germ-free and SPF animals had ad libitum access to food and tap water. Husbandry conditions were equal except for the need to isolate and house germ-free mice in isolators and keep them in sterile conditions. Animals were kept at 21 °C, on a 12 h light/12 h dark cycle, with nesting and environmental enrichment materials supplied to all cages.

Fresh fecal pellets from 2 month old germ-free and SPF mice (*n* = 10, 5 males and 5 females for each group) were collected.

#### 2.2.2. ACE2 Knock-Out Animals

ACE2-deficient mice [35] on the C57BL/6 genetic background were used in the study. Genetic deletion of the *Ace2* gene in these mice results in the lack of ACE2 protein in the gut [36]. Mice were bred and housed at the Max-Delbrück-Center for Molecular Medicine. Animals were kept on a 12 h light/12 h dark cycle, with ad libitum access to food and tap water, with controlled humidity and environmental temperature.

Fresh fecal pellets from 4 month old ACE2-KO mice (*n* = 10, 4 males ACE2^−/y^ and 6 ACE2^−/−^ females) and corresponding wild-type controls (*n* = 13, 4 ACE2^+/y^ males and 9 ACE2^+/+^ females), were collected.

#### 2.2.3. ACE Knock-Out Animals

Total ACE-KO mice were created and maintained in the Animal Facility of the Cedars-Sinai Medical Center (Los Angeles, CA, USA) as previously described [37]. Total ACE-KO were created by mating heterozygous animals, which produced both homozygous total ACE-KO mice and wild-type mice that were used as controls. ACE C-domain KO and ACE N-domain KO mice were created and maintained in the same Animal Facility, as previously described [38,39]. Point mutations in the catalytic region of the ACE C- or N-domain result in the loss of zinc binding and, consequently, the enzymatic activity of the mutated domain. Wild-type mice of the same C57BL/6J background, raised in the same Animal Facility, were used as controls for the ACE C- and N-domain mice. All animals were maintained in individually ventilated cages with controlled humidity and temperature, in a 12 h light/12 h dark cycle, with nesting, and environmental enrichment materials supplied to all cages, with ad libitum access to standard food and tap water.

Fresh fecal pellets from 3–5 month old total ACE-KO mice (*n* = 10, 5 males and 5 females) and corresponding controls (*n* = 9, 4 males and 5 females), as well as 5 month old ACE C-domain KO mice (*n* = 6, 1 male and 5 females), 4–5 month old ACE N-domain KO mice (*n* = 10, 5 males and 5 females) and 4–5 month old corresponding controls (*n* = 10, 5 males and 5 females) were collected.

### 2.3. Feces Collection and Preparation

Fresh fecal pellets were obtained from the cage of each experimental group and used for determining enzyme activity and total protein content.

For ACE and ACE2 activity measurements, freshly collected fecal pellets were homogenized at a ratio of 200 mg of feces to 1 mL of buffer containing 100 mM sodium borohydride buffer, pH 7.2, 340 mM sucrose, 300 mM NaCl and 1 mM phenylmethylsulphonyl fluoride inhibitor (PMSF). A 200 mM stock solution of PMSF was freshly diluted into the homogenization buffer to reach a final concentration of 1 mM immediately before use. Buffer preparation and sample homogenization were carried out on ice. The homogenates were subsequently centrifuged at 1640× *g* for 20 min at 4 °C, and the supernatants were collected and stored at −80 °C until analysis.

For total protein quantification, approximately 60 mg of cage fecal pellets were collected and homogenized in 500 µL of a U9 buffer containing 9 M Urea, 2% CHAPS, 50 mM Tris, protease inhibitor cocktail (cOmplete™, Mini, EDTA-free Protease Inhibitor Cocktail, 1 pill to 10 mL buffer) (all from Merck^®^, Darmstadt, Germany), adjusted to pH 9.0. Homogenization was performed in a 2 mL round bottom tube containing 2.8 mm and 5 mm ceramic beads, using a Precellys Evolution Tissue Homogenizer (Bertin Technologies, Montigny-le-Bretonneux, France). Samples were then incubated for 1 h at room temperature. Afterwards, 500 µL of U1 buffer [(0.9 M Urea, 2% CHAPS, 50 mM Tris, protease inhibitor cocktail (cOmplete™, Mini, EDTA-free Protease Inhibitor Cocktail, 1 pill to 10 mL buffer); pH 9.0] was added, followed by gentle vortexing and centrifugation at 14,500× *g* for 10 min.

### 2.4. ACE and ACE2 Activity Assays

ACE and ACE2 activities in fecal samples were assessed as previously described [2].

ACE is composed of two catalytic domains (C- and N-domain,) which exhibit distinct substrate preferences and physiological roles. The ACE C-domain is predominantly involved in the conversion of Angiotensin I into Angiotensin II, whereas the ACE N-domain preferentially metabolizes Angiotensin 1–7 into Angiotensin 1–5, and other bioactive peptides, like the anti-inflammatory peptide AcSDKP [40]. Based on these domain specificities, ACE activity was assessed using a fluorimetric assay using two synthetic substrates: Hippuryl-His-Leu (h-HL), which is preferentially cleaved by the C-domain, and Z-Phe-His-Leu (Z-FHL), which is metabolized approximately at the same rate by both domains [41,42]. Importantly, it has been reported that the ratio between the rates of hydrolysis of these two substrates (the Z-FHL/h-HL ratio) can be used to infer the functional profile of ACE present in a given biological sample: somatic human ACE, which contains both active domains, has a Z-FHL/h-HL ratio close to 1; enzymatic activity dominated by the N-domain is associated with a higher Z-FHL/h-HL ratio of (approximately) 4.5; whereas C-domain-predominant activity yields a lower ratio, around 0.7 [43].

For the ACE activity assay, 10 µL of the feces homogenates were incubated with 200 µL of assay buffer containing 100 mM potassium phosphate buffer (pH 8.3), 300 mM NaCl, and 0.1 mM ZnSO_4_, together with either 1 mM Z-FHL or 5 mM h-HL. Reactions were carried out at 37 °C for 10 min. Enzymatic reactions were terminated by addition of 1.5 mL of 0.28 M NaOH. Then, samples were incubated with 100 µL of o-phthaldialdehyde (20 mg/10 mL in methanol) to allow binding of o-phthaldialdehyde to the formed peptide His-Leu, forming a fluorescent product. After a 10 min incubation at room temperature, the fluorescence reaction was stopped with 200 µL of 3 N HCl. Samples were then centrifuged at 1000× *g* for 5 min at 4 °C. The amount of hydrolysis product His-Leu was measured fluorometrically (λexcitation = 360 nm; λemission = 465 nm) using SpectraMax Gemini EM microplate reader (Molecular Devices, San Jose, CA, USA).

ACE2 activity was measured by a fluorometric kinetic assay. In brief, 5 µL of feces homogenates were preincubated for 5 min at 37 °C with an assay buffer containing 75 mM Tris, 1 M NaCl, 0.5 mM ZnCl2, 10 µM captopril, pH 6.5, and complete mini EDTA-free [1 pill for 10 mL of buffer], all from Merck^®^, Darmstadt, Germany, in the presence or absence of the selective ACE2 inhibitor MLN-4760 1 µM (Tocris Bioscience, Bristol, UK). The reaction was initiated by adding 20 µM Mca-APK (Dnp) (Cat. No.: BML-P163-0001, Enzo Life Sciences, Inc., New York, NY, USA), an ACE2 substrate. Fluorescence was recorded for 120 min at 2 min intervals using excitation/emission wavelengths of 320/420 nm, using the SpectraMax Gemini EM microplate reader (Molecular Devices, San Jose, CA, USA). Arbitrary units were registered, calculations were done based on a fluorescence standard curve using OmniMMP^®^ fluorogenic control (Cat. No.: BML-P127-0001, Enzo Life Sciences, Inc., New York, NY, USA), and the time point 0 was used as the internal blank.

Total proteins were quantified according to the Bradford method [44], using bovine serum albumin as a standard. ACE and ACE2 activities were presented as nmol/min/mg of total proteins.

To better characterize RAAS enzymatic balance, in addition to protein-normalized activities, ratios between ACE N- and C-domain activities were calculated, as well as the ratios of ACE2 activity relative to ACE N- and C-domain activities.

### 2.5. Statistical Analysis

Data statistical analysis, as well as all graphs within the manuscript, was performed using GraphPad Prism 9 (Graphpad Software, San Diego, CA, USA). No a priori sample size was calculated, as we used feces from the number of animals available in each Animal Facility. No formal outlier test was applied, and no data points were excluded from the analyses; all data was included in the analysis and confounders were not controlled. The experimental unit was considered the individual mouse.

Data were tested for normality using the Shapiro–Wilk test (small sample size) and statistics were applied accordingly. Data were found to have a non-normal distribution. As such, we used the Mann–Whitney U test for comparisons between groups and the Wilcoxon matched-pairs test for paired comparisons within experimental groups. *p* < 0.05 was considered statistically significant.

## 3. Results

**Figure 1:**
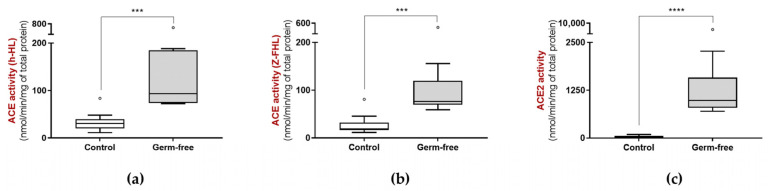
ACE activity using (**a**) h-HL and (**b**) Z-FHL as substrates, and (**c**) ACE2 activity in the feces of 2 month old control (*n* = 10, 5 males and 5 females and germ-free mice (*n* = 10, 5 males and 5 females). *** *p* < 0.001 and **** *p* < 0.0001.

ACE activity (Figure 1a and Figure 1b, respectively) as well as ACE2 activity (Figure 1c) was increased in the feces of germ-free mice compared to that of controls. This suggests that the microbiota may produce molecules that are physiologic inhibitors of ACE and ACE2 activities and/or that the absence of microbiota may increase the intestinal expression of the enzymes, which through shedding increases their fecal activity.

**Figure 2:**
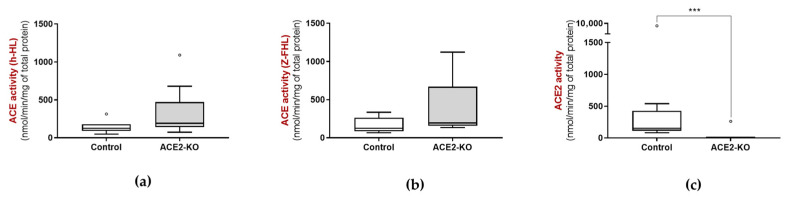
ACE activity using (**a**) h-HL and (**b**) Z-FHL as substrates, and (**c**) ACE2 activities in the feces of 4 month old control (*n* = 13, 4 males and 9 females) and ACE2-KO (*n* = 10, 4 males and 6 females) mice. *** *p* < 0.001.

Additionally, ACE activity was not different between control and ACE2-KO animals (Figure 2a,b), while ACE2 activity was markedly lower in ACE2-KO mice (close to zero) than in controls (Figure 2c). These results suggest that fecal ACE2 activity may be a result of ACE2 shedding from the intestinal epithelial cells.

**Figure 3:**
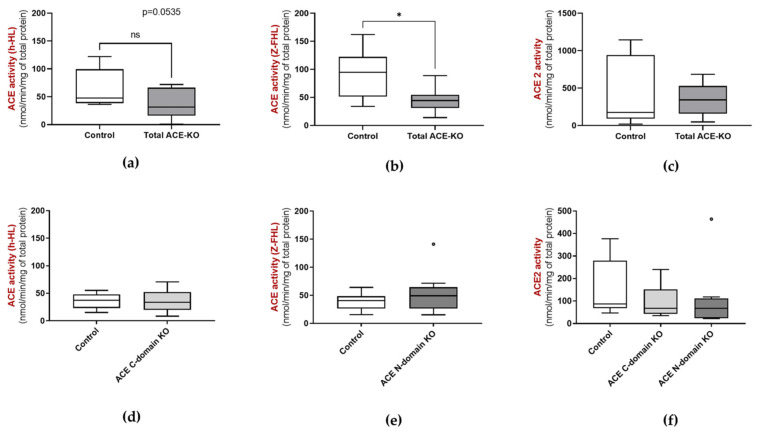
ACE activity using (**a**,**d**) h-HL and (**b**,**e**) Z-FHL as substrates, and (**c**,**f**) ACE2 activity in the feces of 3–5 month old total ACE-KO mice (*n* = 10, 5 males and 5 females) and corresponding controls (*n* = 9, 4 males and 5 females), as well as 5 month old ACE C-domain KO mice (*n* = 6, 1 male and 5 females), 4–5 month old ACE N-domain KO mice (*n* = 10, 5 males and 5 females) and corresponding controls (4–5 month old, *n* = 10, 5 males and 5 females). * *p* < 0.05.

In total ACE-KO mice, ACE activity was lower than that of controls, but not close to zero (Figure 3a and Figure 3b, respectively). This suggests that although intestinal ACE contributes substantially to fecal ACE activity, other additional ACE-like catalytic activity from other sources is present. No differences in ACE (Figure 3d,e) or ACE2 (Figure 3c,f) activities were observed between ACE C- and N-domain KO and controls.

**Figure 4:**
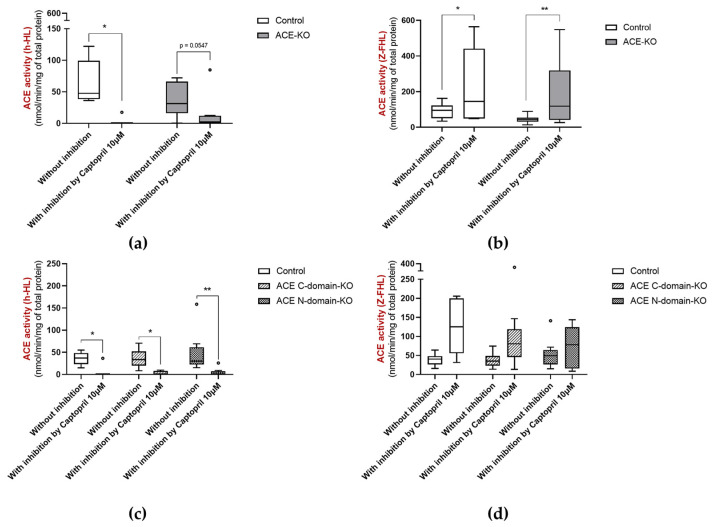
ACE activity using (**a**,**c**) h-HL and (**b**,**d**) Z-FHL as substrates in the feces of 3–5 month old total ACE-KO mice (*n* = 10, 5 males and 5 females) and corresponding controls (*n* = 9, 4 males and 5 females), as well as 5 month old ACE C-domain KO mice (*n* = 6, 1 male and 5 females), 4–5 month old ACE N-domain KO mice (*n* = 10, 5 males and 5 females) and corresponding controls (4–5 month old, *n* = 10, 5 males and 5 females). * *p* < 0.05 and ** *p* < 0.01.

Given that in ACE-KO mice, ACE activity was lower than in controls but not absent, we quantified the ACE activity in the absence and presence of the ACE inhibitor captopril to ensure that ACE activity was in fact from ACE or any ACE-like enzyme, and not other enzymes. Fecal ACE activity using h-HL as a substrate was abolished in the presence of captopril in controls and in all three groups of ACE-KO mice (Figure 4a,c). Fecal ACE activity using Z-FHL as a substrate was increased in the presence of captopril in the feces of ACE-KO mice and corresponding controls (Figure 4b) and showed a tendency to be increased in the presence of captopril in the feces of ACE C- and N-domain KO as well as corresponding controls (*p* > 0.05) (Figure 4d).

**Figure 5:**
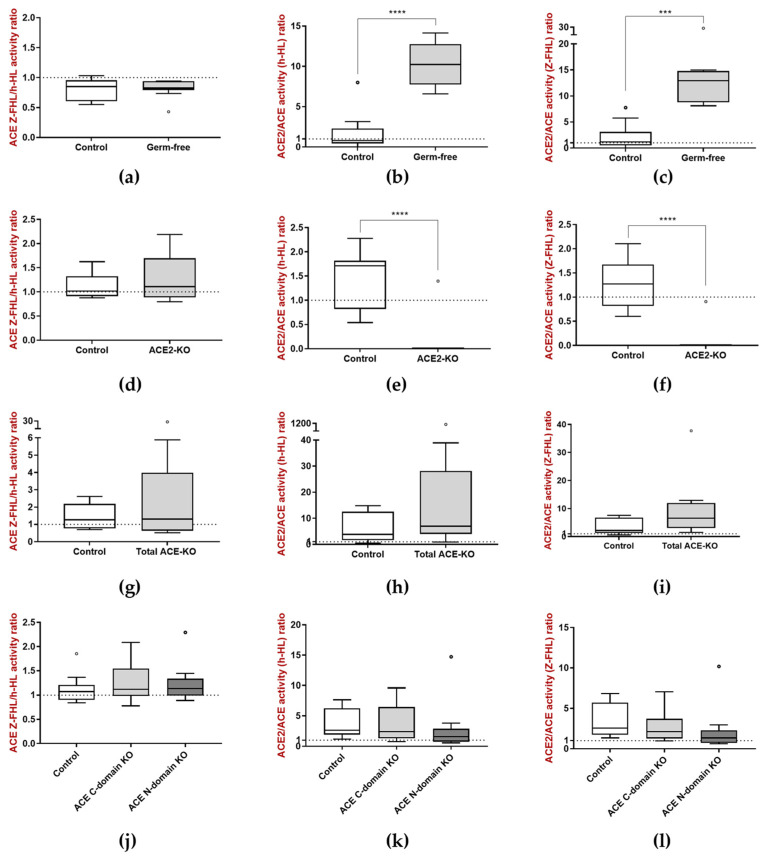
Ratios between (**a**,**d**,**g**,**j**) ACE activity using h-HL and ACE activity using Z-FHL, (**b**,**e**,**h**,**k**) ACE2 and ACE activity using h-HL, and (**c**,**f**,**i**,**l**) ACE2 and ACE activity using Z-FHL in the feces of control and total ACE-KO, ACE C-domain KO and ACE N-domain KO mice. *** *p* < 0.001 and **** *p* < 0.0001.

Ratios between enzymatic activities were also calculated to allow a better understanding of the overall balance between ACE and ACE2 in the feces. ACE Z-FHL/h-HL activity ratio was close to 1 in all groups of mice, with no differences between experimental groups, showing that the N- and the C-domain are equally active in the feces of these animals (Figure 5a,d,g,j). Additionally, the ACE2/ACE activity (h-HL) ratio as well as ACE2/ACE activity (Z-FHL) ratio in the feces of germ-free mice was higher than 1 and higher than that of control animals (which was close to 1) (Figure 5b,c), suggesting that in the feces of both groups of animals, ACE2 activity is higher than ACE activity, and that this difference is accentuated in the feces of germ-free animals. Also, given that in ACE2-KO mice ACE2 activity was close to zero, the ACE2/ACE activity (h-HL) ratio as well as ACE2/ACE activity (Z-FHL) ratio were close to zero in those animals, and decreased compared to that of controls (Figure 5e,f). Finally, no differences were found in the ACE2/ACE activity (h-HL) ratio or the ACE2/ACE activity (Z-FHL) ratio between all three groups of ACE-KO mice and the corresponding controls, although these ratios were higher than 1 in all groups, suggesting higher ACE2 activity compared to ACE activity (Figure 5h,i,k,l).

## 4. Discussion

**Figure 6:**
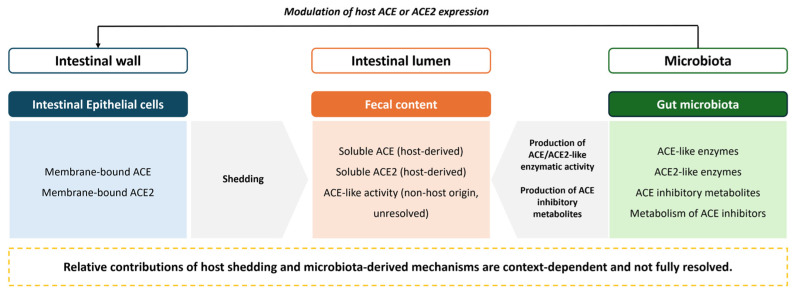
Schematic representation of the potential sources and modulatory pathways underlying ACE and ACE2 activity detected in fecal samples.

Our study is the first to explore the putative origin of ACE and ACE2 activity in the feces. These findings provide new insights on the interplay between intestinal wall shedding and microbiota as sources of fecal ACE and ACE2 activity (Figure 6).

Our results point to host intestinal ACE2 shedding as a major contributor to measurable fecal ACE2 activity, since that activity was almost absent in ACE2-KO mice under the present experimental conditions. It has been reported that ADAM-17, an ACE2 sheddase, is present in the colon [19,20]. Also, studies suggest that the ADAM-17-mediated shed ACE2 ectodomain remains catalytically active [12,45]. So, it is plausible to assume that in the intestine, ADAM-17 can cleave ACE2 in its soluble form that remains active in the feces. However, it has also been reported that the microbiota reduces the intestinal expression of ACE2, since germ-free mice presented with increased ACE2 intestinal expression compared to conventional corresponding rodents [29]. Additionally, colonization of germ-free rats by either co-housing with conventional animals for a period of time [30] or humanization with healthy microbiota [31] reduces intestinal ACE2 expression. This goes in accordance with our results on germ-free mice, where ACE2 activity is increased. One can speculate that germ-free mice present with higher intestinal ACE2 expression, and that shedding of that intestinal ACE2 will lead to increased ACE2 activity in the feces of those animals compared to that of controls, which we found in our study. Nevertheless, it has also been reported that *Paenibacillus* sp. B38-derived ACE2-like enzyme with carboxypeptidase activity suppressed Ang II-induced hypertension, cardiac hypertrophy, and fibrosis in mice [33]. Also, ACE2/ACE activity ratios were close to 1 in controls, but markedly increased in germ-free animals, supporting the idea that the microbiota, by reducing intestinal expression of ACE2, reduces fecal ACE2 activity. Overall, in healthy animals, fecal ACE2 activity seems to result predominantly from host ACE2 shedding, although the microbiota seems to be an important modulator, by regulating (decreasing) the intestinal ACE2 expression, and producing ACE2-like enzymes or (although, to our knowledge, never reported) ACE2 inhibitory metabolites.

Shedding from the intestinal wall also seems relevant as a source of fecal ACE activity, since ACE activity was reduced in the feces of ACE-KO animals compared to their corresponding controls. Shedding of ACE has been widely described [5,6,10,11], although the responsible enzymes have not been fully identified [7,16]. However, although ACE is present in the intestinal wall [1], intestinal shedding of this enzyme remains poorly understood. Similarly to what we discussed for ACE2, the microbiota might also be modulating fecal ACE activity. Nevertheless, the residual ACE activity observed in ACE-KO mice should be interpreted cautiously and cannot be considered direct evidence of a microbial origin. Rather, these findings suggest that additional ACE-like catalytic activity may be present in the intestinal lumen. Such activity could reflect microbiota-derived ACE-like activity, which has been previously reported in a *Xanthomonas axonopodis* pv. *citri*-derived ACE-like protein [32], or possible hydrolysis of the synthetic substrates by other host or luminal peptidases, indirect effects of microbiota-derived metabolites, or changes in substrate availability within the fecal matrix. This goes in accordance with our data on germ-free animals that showed higher fecal ACE activity when compared to controls. These findings suggest that the microbiota may normally reduce measurable fecal ACE activity, potentially through production of ACE inhibitors, which have been associated with the genera *Lactobacilus* and *Bifidobacterium* [22,23,24,25]. Interestingly, the microbiota itself can also catabolize ester ACE inhibitors, like quinapril [27], further supporting a complex interaction between microbiota-derived metabolism and RAAS-related pathways. Our results on ACE C-domain and N-domain KO mice are also in agreement with this multifactorial interpretation. These genomic mutations on these animals lead to depletion of ACE’s zinc binding domain [41], which is essential for ACE activity, yet measurable fecal ACE activity remained detectable. Additionally, captopril abolished hydrolysis of h-HL, confirming that the activity being measured with this substrate in our assay was ACE-dependent. In contrast, inhibition with captopril increased the ACE activity using Z-FHL as a substrate, suggesting that Z-FHL hydrolysis in fecal homogenates may involve additional captopril-insensitive enzymatic pathways that become more evident after ACE inhibition, like cleavage of Z-FHL by other host- or microbiota-derived peptidases, altered substrate competition after ACE blockade, or matrix-related effects on substrate accessibility and product accumulation in the fecal homogenate. Regardless of the relative contribution of shedding or the microbiota, neither mechanisms appeared to substantially alter the balance between ACE catalytic domains, as the ACE Z-FHL/h-HL activity ratio remained close to 1 in all groups, which represents an equilibrium between the two ACE catalytic domains [43].

One limitation to our study is the fact that animals from the three different groups (germ-free and ACE-KO and ACE2-KO) were housed in different animal facilities, which could contribute to a different microbiota population within groups. Because housing environment, diet, sanitary barrier conditions, and colony-specific microbial composition are known to influence gut microbiota structure and host physiology, these differences may have introduced confounding variability across different groups. For this reason, the different mouse lines were not intended for direct quantitative comparison with one another, and each model was interpreted only in relation to its own corresponding control group, maintained in the same facility and with a similar genetic background. Also, animals were age-matched within each experimental comparison, and both sexes were represented in most groups; however, the study was not specifically powered to detect sex-dependent effects. In addition, although previously characterized ACE-KO lines were used, direct confirmation of intestinal ACE expression or tissue ACE activity was not performed in the same animals used for fecal assays. Therefore, residual host-derived catalytic activity cannot be fully excluded. Future studies combining standardized housing conditions, controlled microbial colonization strategies, and direct microbiota profiling, coupled with direct validation of host intestinal ACE/ACE2 expression/activity in the same animals, will be necessary to further dissect host and microbial contributions.

## 5. Conclusions

Our study shows that fecal ACE and ACE2 activity is a result of combined intestinal shedding and non-host luminal mechanisms, possibly microbiota-associated. The microbiota may influence fecal ACE and ACE2 activity through multiple non-exclusive mechanisms, including modulation of intestinal enzyme expression and the production of metabolites with inhibitory potential or ACE-like enzymatic activities. Understanding the source of these enzymes in the feces is of the upmost importance because it adds to our knowledge of the physiology of the gastrointestinal tract and expands our current understanding of how the gastrointestinal environment contributes to local regulation of the RAAS.

## Data Availability

Data is available upon reasonable request to the corresponding author.

## Abbreviations

The following abbreviations are used in this manuscript:

ACE	angiotensin-converting enzyme
ACE2	angiotensin-converting enzyme 2
h-HL	Hippuryl-His-Leu
KO	knockout
RAAS	renin–angiotensin–aldosterone system
SPF	specific-pathogen-free
Z-FHL	Z-Phe-His-Leu
